# Human Cell Modeling for Cardiovascular Diseases

**DOI:** 10.3390/ijms21176388

**Published:** 2020-09-02

**Authors:** Melania Lippi, Ilaria Stadiotti, Giulio Pompilio, Elena Sommariva

**Affiliations:** 1 Unit of Vascular Biology and Regenerative Medicine, Centro Cardiologico Monzino IRCCS, 20138 Milan, Italy; mlippi@ccfm.it (M.L.); istadiotti@ccfm.it (I.S.); gpompilio@ccfm.it (G.P.); 2 Department of Clinical Sciences and Community Health, Università degli Studi di Milano, 20122 Milan, Italy

**Keywords:** human cell model, cardiovascular disease, disease modeling, heterologous system, primary cells, embryonic stem cells, human induced pluripotent stem cell, co-cultures, engineered 3D tissue

## Abstract

The availability of appropriate and reliable in vitro cell models recapitulating human cardiovascular diseases has been the aim of numerous researchers, in order to retrace pathologic phenotypes, elucidate molecular mechanisms, and discover therapies using simple and reproducible techniques. In the past years, several human cell types have been utilized for these goals, including heterologous systems, cardiovascular and non-cardiovascular primary cells, and embryonic stem cells. The introduction of induced pluripotent stem cells and their differentiation potential brought new prospects for large-scale cardiovascular experiments, bypassing ethical concerns of embryonic stem cells and providing an advanced tool for disease modeling, diagnosis, and therapy. Each model has its advantages and disadvantages in terms of accessibility, maintenance, throughput, physiological relevance, recapitulation of the disease. A higher level of complexity in diseases modeling has been achieved with multicellular co-cultures. Furthermore, the important progresses reached by bioengineering during the last years, together with the opportunities given by pluripotent stem cells, have allowed the generation of increasingly advanced in vitro three-dimensional tissue-like constructs mimicking in vivo physiology. This review provides an overview of the main cell models used in cardiovascular research, highlighting the pros and cons of each, and describing examples of practical applications in disease modeling.

## 1. Introduction

Cardiovascular diseases (CVDs) include a group of disorders affecting heart and blood vessels and are the main cause of death worldwide: only in 2016, ~17.6 million deaths were attributed to CVDs, with an increase of 14.5% from the past decade [1]. A number of genetic and environmental factors intervenes in the onset and progression of CVDs pathogenesis, including structural, electrical, and metabolic alterations, cells death, inflammation, fibrosis, and tissue remodeling. The comprehension of the underpinned molecular mechanisms is the key for therapeutic advancements. Therefore, reliable disease models for the investigation of the molecular basis of CVDs are an emergent necessity, in order to recapitulate in vitro what happens in the human organism [2]. The refinement of in vitro models is also fueled by public advocacy to minimize the use of animals in research laboratories. Moreover, the investigation on animal models is compelled by compromises between economic and technical convenience of simpler organisms and the greater resemblance to humans of larger animals. As an example, mice have been widely used as a model of CVDs, due to the easy maintenance in laboratory and the relatively high homology with human genome, even if their cardio-circulatory system does not perfectly retrace human physiology [3]. Therefore, human cell models, easily obtainable, unlimitedly available, recreating the cellular and extracellular niche of heart and vessels and exhibiting prolonged viability and correct physiology, would be an ideal alternative model to retrace the onset and the progression of CVDs and to test novel therapeutic strategies. In the present review, the main human cellular types used in cardiovascular research are described ranging from the simplest to the most complex three-dimensional constructs that best recapitulate the real physiology of the human native tissue.

## 2. Non-Cardiovascular Cells

Despite their derivation, non-cardiovascular cell models can provide clues to cardiovascular research, using methods that are affordable or easily applicable. Generally, these models are cell lines utilized to express specific genes or easily accessible primary cells obtainable for large population studies [4]. A cloned protein can be expressed in cells in which it is normally not present, the so-called heterologous systems. The use of such simple systems facilitates the study of the protein function, avoiding the issue of the complex regulations, which it is endogenously subjected to. Heterologous systems have always been used in cardiovascular research, because of both the limited availability of human myocardial samples and the need of a simpler model than the native tissue. As for cardiac genetic diseases is concerned, pathological mutations have often been found in genes encoding junctional proteins, cytoskeletal components, signaling molecules, ion channels and accessory proteins. In most cases, heterologous systems allowed the heterologous expression of those proteins, either Wild Type (WT) or mutant, for a functional evaluation of the genetic alterations [5].

### 2.1. HEK 293

HEK 293 is the most used heterologous model in cardiovascular field. It is an immortalized cell line that origins from human embryonic kidney with very attractive characteristics for biology research, such as ease of maintenance and high transfection efficiency [5]. Given the low expression of native cardiac channels, the overexpression of exogenous ion channels in HEK 293 allows electrophysiological studies by whole-cell patch clamp recordings, in order to characterize the resulting currents or to investigate specific diseases. In 2009, Milanesi et al. evaluated the functional effect of a mutation in the cardiac pacemaker channel associated with a familial bradycardia, transfecting human hyperpolarization-activated cyclic nucleotide-gated channel 4 (HCN4) into HEK 293. The negative shift of the activation curve found in the heterologous system reflected the slowing of heart rate observed in patients [6]. HEK 293 cells were also used for the study of the functional importance of HCN4 localization on the membrane, in particular on lipid rafts, whose lack influenced the channel kinetics, just like in cardiac tissue [7].

Besides electrophysiology, HEK 293 are used for a wide range of assays for the study of CVDs. A clear example is the work by Asimaki et al., in which they characterized the S39_K40insS mutation in the gene encoding for plakoglobin (PG) associated with arrhythmogenic cardiomyopathy (ACM), through the transfection of the mutant gene into this cell line. Western Blot analysis, cell-proliferation assay, caspase-3 assay, confocal microscopy, and electron microscopy suggested that S39_K40insS mutation altered the properties of cell junctions and the regulatory mechanisms mediated by Wnt-signaling pathway [8]. In 2013, Cannavo and colleagues transfected the β1-Adrenergic Receptor (β1AR) and the Sphingosine-1-Phosphate Receptor 1 (S1PR1) into HEK 293 to evaluate the direct interaction between these two receptors: the treatment with the respective agonists isoproterenol and Sphingosine 1-phosphate highlighted the reciprocal regulation between the two receptors, mediated by the activation of G protein-coupled receptor kinase-2, and the beneficial effects of S1PR1 in the offsetting of injurious β1AR overstimulation in heart failure [9].

### 2.2. Buccal Mucosa Cells

Buccal mucosa cells (BMCs) are stratified non-keratinizing epithelial cells, easily and safely obtainable from the inside of the cheek. This is not a classic heterologous model for cardiovascular research, but due to their epithelial origin, they express desmosomes and gap junctions. Asimaki et al. compared the distribution of cell junction proteins in BMCs derived from ACM patients and healthy controls. The desmosomal protein PG and the major cardiac gap junction protein Connexin 43 (Cx43) resulted markedly decreased in BMCs from ACM patients, similarly to what happens in the heart. In the same work, the incubation of ACM BMCs with SB216763, a glycogen synthase kinase 3β (GSK3β) inhibitor that reactivates Wnt pathway, seemed to restore PG and Cx43 distribution, showing the promising role of these cells as a drug screening model for ACM [10]. Another application of BMCs in CVDs was described by Wong et al., who measured the telomere length in different cell types from ischemic heart failure (IHF) patients and their offspring, in order to assess a familial predisposition and a correlation between a shorter telomere and the pathogenesis of the disease. Among the analyzed cells, including BMCs, only leukocytes showed a shorter telomere in patients and in their healthy offspring compared to controls, suggesting that only selected cell types are involved in the potential mechanisms linking telomere length and IHF and in its inheritance pattern [11].

## 3. Cardiovascular Cells

The complex and hierarchical architecture of the cardiovascular system is composed by numerous cell types, which all together contribute to the correct functioning of the entire apparatus. In the heart, the combined action of cardiomyocytes (CMs) and non-myocyte cells orchestrate the cardiac physiology. In vessels, endothelium and smooth muscle play distinct roles, both crucial for their correct functioning. Single cell type dysfunction may lead to the development of different pathologies. The most intuitive method to characterize a pathology is to directly analyze the affected organ and therefore to use a human primary culture. Primary culture are composed of cells directly isolated from the native tissue. This is, however, not always possible, especially in the cardiovascular system, where primary cells are poorly accessible. Their obtainment may normally occur during surgery or organ transplantation. In addition, primary cells do not last long in culture, especially when the proliferative potential is close to zero, as CMs. To solve this problem, some researchers developed immortalized post-mitotic lines of CMs, in order to create an unlimited resource of cardiac cells.

### 3.1. Primary Atrial and Ventricular Cardiomyocytes

Atrial and ventricular CMs are striated muscle cells, capable of contracting, thanks to the electrical impulse received from the conduction system of the heart, triggering the action potential. They form a functional syncytium through gap junctions, allowing a synchronous contraction of the muscle [12]. Primary CMs are considered the most representative model of functional cardiac cells in vitro, but they are poorly available and hard to maintain in culture; their isolation from atrial appendage resection or rare ventricular biopsies is usually performed by enzymatic digestion, not always successful. The most studied cardiac disease with human CMs is terminal heart failure, because of the accessibility of explanted hearts. However, these cells often represent the final stage of a disease and severe post-explant remodeling may occur, thus, masking the pathogenic causes. Instead, a better understanding of the pathogenic mechanisms would be obtained by analyzing cardiac cells from patients at an earlier stage of diseases [13]. In 1993, Beuckelmann et al. noticed a decrease in the inward rectifier K^+^ current (I_K1_) and in the transient outward K^+^ current (I_to_) densities in ventricular myocytes isolated from patients with terminal heart failure undergoing transplantation, finding a partial explanation of action potential prolongation observed in these subjects [14]. Another work described the use of antisense oligonucleotides to investigate the role of the potassium voltage-gated channel K_V_1.5 in ultra-rapid delayed rectifier K^+^ current (I_KUR_) and I_to_ in atrial myocytes, derived from patients undergoing aortocoronary bypass surgery, a procedure that requires the transection of left atrial appendage, the most common source of atrial tissue. In presence of antisense oligonucleotides against K_V_1.5 coding sequence, atrial cells showed a marked reduction in I_KUR_, but not in I_to_, implying that the channel is essential for the expression of I_KUR_ and does not participate to I_to_ conductance [15]. In 2014, Coppini et al. developed a protocol to isolate myocytes from biopsies of ventricular myocardium from cardiac surgical patients, using a new digestion device, which allowed a lower use of enzymes for single cell separation, with gentle mechanical agitation of the samples. Cells isolated with this novel method were characterized and show regular sarcomeric organization, calcium transients, and electrophysiological responses [13]. The same research group characterized ventricular CMs isolated from patients with obstructive hypertrophic cardiomyopathy (HCM) and used them as an in vitro model to study the molecular mechanism of disopyramide. This molecule is a potent negative inotrope administered to decrease left ventricular outflow tract obstruction in subjects with obstructive HCM. Results showed that disopyramide inhibited ryanodine receptor (RyR), Na^+^, Ca^++^, and K^+^ currents, decreased action potential duration and frequency of arrhythmic afterdepolarization, suggesting an additional antiarrhythmic action [16,17].

### 3.2. Primary Cardiac Mesenchymal Cells

Human mesenchymal stromal cells (MSCs) are adult multipotent cells characterized by three minimal criteria: adherence to plastic; specific surface antigen expression (CD105^+^, CD73^+^, CD90^+^, CD45^−^, CD34^−^, CD14^−^, CD19^−^, and human leukocyte antigen–DR isotype (HLA-DR^-^)); multipotent in vitro differentiation potential to osteoblast, adipogenic and chondrocyte lineages [18]. The stromal fraction of different tissues, such as bone marrow, adipose tissue, placenta, and heart, can be source of MSCs. In the adult heart, cardiac mesenchymal stromal cells (C-MSCs), often simply named cardiac fibroblasts, are abundant and contribute to the normal myocardial structure and function [19,20]. These cells have a key role in cardiac repair, orchestrating the fibrotic remodeling during pathological conditions, migrating into the scar and participating in wound healing [21,22,23]. Primary C-MSCs can be isolated from different human adult cardiac districts, as auricle and right ventricle, digested with collagenase, and expanded in vitro for several passages. Owing their contribution in cardiac homeostasis, isolated C-MSCs served as models in the study of cardiac pathologies that may not directly or exclusively involve CMs [4]. In 2017, C-MSCs were used to investigate the role of High Mobility Group Box 1 (HMGB1) in cardiac remodeling after myocardial infarction (MI), since this protein, which exists in two redox forms, regulates tissue repair after injuries. Researchers found that the non-oxidizable mutant HMGB1 efficiently promoted C-MSCs migration, which affects cardiac remodeling after MI, as demonstrated by in vivo experiments. These results underlined the importance of HMGB1 redox state in inflammatory response and tissue repair [24]. In 2016, the participation of C-MSCs in the adipose substitution of the myocardium occurring in ACM was demonstrated. After confirming the expression of desmosomal genes linked to ACM in C-MSCs, researchers found that ACM C-MSCs were more prone to differentiate in adipocytes compared to controls, given the earlier and higher lipid accumulation and the enhanced expression of adipogenic genes. These findings showed that C-MSCs contribute to ACM phenotype, a disease previously considered confined to contractile cells. C-MSCs became a novel promising in vitro model for future mechanistic ACM studies [25].

#### 3.2.1. C-Kit Positive Cardiac Mesenchymal Cells

Different papers have been published on a subpopulation of C-MSCs, which express the c-kit marker. C-kit (KIT proto-oncogene receptor tyrosine-protein kinase) has long been considered a progenitor marker since the receptor binding to the ligand “stem cell factor” initiates a signaling cascade leading to cell survival, proliferation, and differentiation [26]. The resident c-kit^+^ cell population in the heart has been mainly recognized as endothelial progenitor cells, with self-renewing and multipotent cardiogenic properties [27,28]. Within the cardiac resident c-kit^+^ population, the proportion isolated with C-MSCs show phenotypical and functional characteristics in common with them, but they represent only the 0.5–1.5% of these cells [22,29]. There are numerous controversies concerning c-kit^+^ C-MSCs classification and nomenclature, but their impact on cardiac homeostasis as well their cardiac regenerative potential has been demonstrated [30]. Recently, Gambini et al. analyzed the differentiation properties of cardiac mesenchymal progenitor cells (CMPCs, stromal cells positive for c-kit) deriving from patients with Atrial Fibrillation (AF), in order to establish the pro-fibrotic contribution of these cells in the pathogenesis of the disease, where atrial fibrosis has a critical role. Cells were isolated from patients’ atrial appendages and AF CMPCs resulted fewer in number and less capable of expansion than controls, but with a more pronounced pro-fibrotic phenotype [31].

#### 3.2.2. Fibro/Adipocyte Progenitors

Another subset of MSCs is represented by cells positive for Platelet-Derived Growth Factor Receptor α (PDGFRα), the Fibro/Adipocyte Progenitors (FAPs), mainly known for their role in fibrosis and adipogenesis occurring in skeletal muscular dystrophies [32]. FAPs express both adipogenic and fibrogenic markers and have also been identified in the mesenchymal compartments of the heart, becoming putative candidates as source of both adipocytes and fibrosis in pathological conditions, such as ACM [33,34]. Indeed, in 2016, Lombardi et al. isolated and characterized human FAPs, confirming their bipotential commitment. In order to investigate a role of FAPs in ACM, researchers verified the expression of desmosome proteins, found only in adipogenic but not in fibrogenic subpopulations [35]. Experiments on mice revealed that the inhibition of FAPs fibrogenic differentiation could be a therapeutic strategy after myocardial infarction, but further studies on human models are necessary [36].

### 3.3. Primary Endothelial Cells

Endothelium is the inner face of blood vessels and plays a crucial role in vascular wall function, regulating blood pressure, autocrine and paracrine signaling, adhesion and transmigration of inflammatory cells. A dysfunction in endothelial cells (ECs) can represent an initial step in the pathophysiology of several CVDs. This primarily occurs through altered adhesion molecules expression, which leads to an inflammatory condition, facilitating the onset of pathologies such as atherosclerosis [37]. Endothelial primary cells have a finite life span and their availability is relatively limited. Nevertheless, over the years, primary ECs have been isolated from different sources, including vascular biopsies and umbilical cord. The human umbilical vein endothelial cells (HUVECs) have been the most used in different investigations [38]. As an example, HUVECs served as a model to study the implication of microRNA-133 (miR-133) in the pathogenesis of angiotensin II (Ang II)-dependent hypertension. The group discovered that increasing concentrations of Ang II inhibited miR-133 expression and that a downregulation and an upregulation of miR-133 suppressed and enhanced HUVECs viability, respectively. They hypothesized the target of miR-133 being pro—renin receptor (PRR), whose silencing decreased Ang II-induced apoptosis [39]. Since arterial and venous ECs have different embryonic origins and show distinct molecular and functional identities, it can be opportune to choose accurately the endothelial subtype to model a CVD [40]. For example, in 2005, Deng et al. investigated the molecular cause of the different susceptibility to atherosclerosis between arteries and veins, finding that the basal gene expression pattern of saphenous vein ECs exhibit a higher protection against endothelial dysfunctions compared to coronary artery ECs, hinting arterial cells as a better model to study atherosclerosis. [41]. In fact, atherosclerosis is a chronic inflammatory pathology, which mainly affects artery and seldom vein walls, characterized by lipid accumulation and activation of endothelium, promoting adhesion and infiltration of inflammatory cells. In 2018, Fan et al. evaluated the role of mammalian Target of rapamycin (mTOR) pathway in atherosclerosis using human aortic endothelial cells (HAECs): inhibition of mTOR decreased vascular cell adhesion protein 1 (VCAM1) expression and consequently monocyte adhesion in inflammatory condition, mimicked by the exposure of HAECs to tumor necrosis factor-α (TNF-α). The researchers individuated the molecular mechanism, finding that mTOR inhibition allowed the activation of protein kinase C-α (PKC-α) and the upregulation of miR-200-3p, which blocked *VCAM1* transcription through the downregulation of the transcription factor GATA6 [42]. Another work described the use of proximal pulmonary arterial ECs for the study of C-reactive protein (CRP) in chronic thromboembolic pulmonary hypertension (CTEPH). ECs from CTEPH patients showed an overexpression of LOX1, a CRP receptor, and this probably led to a higher activity of CRP. Increasing concentrations of CRP induced the expression of intercellular adhesion molecules 1 (ICAM1), promoting monocyte adhesion to ECs, and enhanced the secretion of von Willebrand factor, contributing to the persistent obstruction of proximal pulmonary arteries in CTEPH patients [43].

### 3.4. Primary Vascular Smooth Muscle Cells

Vascular smooth muscle cells (VSMCs) are important players in blood vessel physiology. They are placed in the *tunica media* of vessels and are responsible for their contraction and relaxation. In healthy adult blood vessels, VSMCs do not proliferate and show a differentiated contractile phenotype. In pathological conditions or injury, VSCMs lose their quiescent phenotype to assume a synthetic phenotype, characterized by proteosynthesis, migration, and growth activities. These features promote repair, but may degenerate in thickening of blood vessel wall in a hypertensive situation, atherosclerotic plaques formation, or vascular calcification [44]. Primary human VSMCs are a limited resource, obtainable from biopsies of patients undergoing aortic or pulmonary artery surgery. As for ECs, the choice of the VSMC subtypes to model some CVDs can be relevant, since, despite exhibiting several common characteristics, arterial and venous cells have distinct embryonic origin and are subjected to different hemodynamic forces [45]. A clear example is the work of Deng et al., who compared genetic profiles and proliferative and migratory responses of smooth muscle cells from saphenous vein (SVSMs) and from coronary artery (CASMs) to platelet-derived growth factor (PDGF) and oxidized low-density lipoproteins (LDL). SVSMs showed a higher expression of atheroprotective genes than CASMs in a basal state, which explained the atheroma-resistance in normal anatomic conditions; SVSMs responded more markedly to atherogenic stimuli, suggesting that different transcription patterns of arteries and veins contribute to susceptibility to atherosclerosis, besides pressure and flow factors [46]. Atherosclerosis is a condition leading VSMCs to proliferation and apoptosis. A key regulator of this process is the long non-coding RNA LOC285194, a non-protein-coding transcript whose overexpression reduces the proliferation and promotes the apoptosis of human aortic (HA) VSMCs, while its inhibition stimulates proliferation. Furthermore, downregulation of LOC285194 induces HA-VSMCs migration and invasion, making the long non-coding RNA a good therapeutic target candidate in atherosclerosis treatment [47]. VSMCs have also been used by Quarck et al. as a model for CTEPH, associated with proximal pulmonary artery obstruction and vascular remodeling. Analysis of migration and proliferation revealed that CTEPH pulmonary artery smooth muscle cells (PASMCs) showed a significant increase in these activities, compared to controls. These results, coupled with similar data obtained in pulmonary arterial endothelial cells, demonstrated a contribution of different vascular cell types to vascular remodeling in CTEPH patients [48]. The enhanced migration in CTEPH PASMCs was then demonstrated to be mediated by renin-angiotensin system (RAS). Indeed, Zhang et al. found that Ang II helped this process through the activation of Ang II receptor and phosphorylation of extracellular signal-regulated kinase ½ (ERK1/2). With these results, it was possible to speculate on molecules targeting RAS as a therapy for CTEPH [49].

### 3.5. Cell Line AC 16

AC 16 is an immortalized cell line, deriving from primary human ventricular CMs fused with SV40 transformed human skin fibroblasts [50]. The resulting cells are stable, have been passaged for over 120 generations, and can switch from a proliferative to a more differentiated state, depending on culture conditions. AC 16 cells express several cardiac-specific transcription factors (GATA4, MycD, and Nuclear Factor of Activated T cells 4), contractile proteins (troponin 1, α- and β-Myosin Heavy Chain, α-cardiac actin, α-actinin), junctional proteins (desmoplakin, ventricular Myosin Light Chain 1, Cx40, Cx43), and show coupled gap junctions. By silencing SV40 T-antigen or altering culture conditions, AC 16 stop to proliferate and reach a more differentiated quiescent state, characterized by bone morphogenetic protein 2 (BMP2) expression, and forms a multinucleated syncytium. These CMs seem to be at a pre-contractile stage; indeed, their myofibrils are not organized in sarcomeres, T-tubules are absent, no evoked action potential are produced by depolarizing pulses, and major inward and outward currents are missing [50]. Recently, AC 16 cells treated with isoproterenol hydrochloride (Iso) were used as a cardiac hypertrophy model: Iso induced an increase in the size of the cells and the activation of cardiac hypertrophy markers, such as atrial natriuretic peptide (ANP), B-type natriuretic peptide (BNP) and β-myosin heavy chain (β-MHC). The aim of the work was to test the anti-inflammatory effects of pyrroloquinoline quinone (PQQ), which resulted to attenuate the activation of nuclear factor k-light-chain-enhancer of activated B cells (NF-κB) phosphorylation, to reduce the Iso-induced accumulation of reactive oxygen species (ROS), to inhibit the expression of hypertrophy markers and to increase the level of matrix metalloproteinase (MMP) in cardiac hypertrophic AC 16 cells [51]. Another work proposed AC 16 CMs as an ischemia/reperfusion (IR) injury model to evaluate the cytoprotective role of yes-associated protein 1 (YAP1), the main effector of Hippo-signaling pathway. After simulated IR injury using a hypoxic chamber, AC 16 cells overexpressing YAP1 showed a reduction in apoptosis, hypertrophy, and generation of ROS, hinting to the potentiality of YAP1 as a therapeutic target after myocardial infarction [52].

## 4. Stem Cells

Because of the restrictions in primary cells use and the distance of heterologous systems from original tissue, the isolation of human pluripotent stem cells has captured the interest of many researchers in numerous fields. The ability of proliferating indefinitely and differentiating into most cell types, including CMs, has given a unique opportunity to obtain an unlimited source of cardiovascular cells in a dish. Stem cells have enormously contributed to elucidate numerous mechanisms that guide embryonic development and have represented an innovative research platform that meets the needs of disease modeling, drug discovery, and regenerative therapy.

### 4.1. Human Embryonic Stem Cells

Embryonic stem cells (ESCs) were isolated for the first time in 1981 from the inner cell mass of a mouse blastocyst by Evans and colleagues [53]. The crucial characteristics of ESCs are early embryonic derivation, indefinite and undifferentiated proliferation, and potential ability to differentiate into mature cell types from all three germ layers. Some years later, in 1998, Thomson et al. could obtain human embryonic stem cells (hESCs) [54]. Because of their origin from human embryos produced by in vitro fertilization, ethical and practical problems concern hESCs. hESCs express high level of telomerase activity, the pluripotency markers octamer-binding transcription factor 4 (Oct-4), sex determining region Y-box 2 (Sox2), and NANOG, and cell surface markers in common with non-human primate ESCs, such as stage-specific embryonic antigen 4 (SSEA-4), Tra 1-60 and Tra 1-81 [54,55].

The differentiation potential of hESCs promoted the development of protocols for the commitment toward various cardiovascular cell types, such as CMs, ECs, and SMCs. Both hESC-ECs and hESC-SMCs closely look like their in vivo counterparts; in particular, hESC-ECs can spontaneously organize in vitro vessel-like structures in a pattern that recapitulates embryonic vascularization, showing the potential therapeutic implications, among which repair of ischemic tissues and tissue engineering of vascular grafts [56,57,58]. The possibility to induce the cardiac differentiation of hESCs provided the first source of human heart cells for large-scale in vitro experiments. Three main general approaches have been defined to generate CMs from hESCs: (i) embryoid bodies formation, a differentiation through spontaneous cell aggregation, which resembles early steps of in vivo development; (ii) hESCs and stromal cells co-culture, a visceral endoderm-like, which directs differentiation with the endoderm influence, as during embryogenesis; (iii) monolayer culture supplemented with specific growth factor and small molecules inducing mesodermal and cardiac commitment [59]. hESC-CMs express cardiac structural proteins (tropomyosin 1 and 2), several actin and actin-regulatory proteins (β-actin, α-actinin, coronin), vascular collagens (COL8A1, COL6A3, COL4A3, and 4, COL2A1) and gap junctions; they display stable pacemaker activity, functional syncytium, and conduction properties; electrophysiological recordings detected all the three action potential shapes found in adult heart, thus nodal-, atrial- and ventricular-like, and most ion currents that compose them [60,61]. The mixture of different CMs can represent a limit for modeling diseases that specifically affect only one subtype. Some reports suggest that hESC-CMs can be directed towards ventricular or atrial phenotype modulating the retinoic acid signaling, whereas inhibition of neuregulin (NRG)-1b/ERBB pathway enhances the ratio of nodal-like cells [62,63].

Several areas of cardiovascular research have been incredibly advantaged by the use of hESCs: development studies, drug discovery and regeneration therapy. Moreover, some experiments have used hESC-CMs as disease models. In 2011, Földes et al. differentiated hESCs in CMs to study HCM. They exposed hESC-CMs to different hypertrophic stimuli and generated a model of the pathology, characterized by an increase in cell size, reorganized sarcomeres, cytoskeletal rearrangement. In HCM, the alteration of sarcomere structure and functions instigates a cascade of molecular events, including expression and activation of Ca^++^-sensitive and stress-responsive molecules or trophic and mitotic factors, such as ERK, c-Jun-N-terminal kinase (JNK), Ca^++^/calmodulin-dependent protein kinase II (CaMK II), mTOR, calcineurin and p38 mitogen-activated protein kinase (p38-MAPK). Blockers of these kinases partially or completely restored the hypertrophic phenotype in hESC-CMs, revealing their significant role in HCM. Furthermore, a long-term culture of hESC-CMs induced spontaneous cell growth, repressed by p38-MAPK inhibition and enhanced by the constitutive activation of its upstream activator, clear evidence of an implication of this pathway in cell growth [64]. Another approach in HCM studies was carried out by Song and colleagues. More precisely, they exploited CRISPR/Cas9 to knock out α-galactosidase A gene (*GLA*) in hESC line, in order to obtain Fabry disease (FD)-associated HCM CMs. In FD, loss of GLA results in left ventricular hypertrophy due to globotriaosylceramide (Gb3) deposition, with mechanisms still to be elucidated. Like in FD-HCM, these hESC-CMs were characterized by accumulation of Gb3, increase in cell size, expression of several fetal proteins, such as ANP and BNP, and altered balance in MYH6/MYH7 ratio. Furthermore, several proteins involved in vesicle secretion, including Rab GTPases, are downregulated in *GLA*-null hESC-CMs compared to controls and both autophagy and exosome secretion resulted impaired, causing an increase of ROS and consequently apoptosis and necrosis. Therefore, this model recapitulated the typical phenotype of FD-HCM and laid the foundation for development of novel therapies by targeting Rab GTPases to restore vesicle trafficking [65].

### 4.2. Human Induced Pluripotent Stem Cells

In 1986, Gurdon and colleagues demonstrated that nuclei of terminally differentiated adult cells hold all the genetic information to generate an entire organism [66]. These studies represented a turning point for stem cell research: in 2006, Takahashi and Yamanaka identified four essential transcription factors for pluripotency maintenance (Oct3-4, Sox2, c-Myc, and Krüppel-like factor 4), which allowed to reprogram both murine and human fibroblast genome to obtain cells with characteristics similar to ESCs, the so-called induced pluripotent stem cells (iPSCs) [67,68]. Human iPSCs (hiPSC) are self-renewing, able to grow with indefinite lifespan and to differentiate in the three germ layers; however, unlike hESCs, they are easily available, usually from skin fibroblasts or peripheral blood mononuclear cells, and have the advantage of being patient- and pathology-specific [69]. These features make hiPSCs a potential unlimited source of any desired differentiated cell that serves as an in vitro model mainly for genetic diseases, in terms of discovery of molecular mechanisms and of personalized medicine.

#### 4.2.1. Human Induced Pluripotent Stem Cells-Derived Cardiomyocytes

For CVDs investigations, hiPSCs can be differentiated in different cardiac cell types, among which CMs, without the complications concerning biopsies and hESCs. There are three main methods to obtain cardiac commitment in vitro, the same of hESCs: embryoid bodies, to resemble initial phases of development; monolayer cultures, which allow a better access of nutrients and growth factors; co-cultures with visceral endoderm-like cells, secreting procardiac cytokines [70]. Human iPSC-derived CMs (hiPSC-CMs) are spontaneous beating cardiac cells with an immature profile: they differ from adult CMs for several parameters, such as organization of sarcomeres, distribution of gap junctions, electrophysiological properties and co-expression of nodal-, ventricular- and atrial- markers [71,72,73]. As for hESC-CMs, the modulation of retinoic acid drove to atrial or ventricular phenotype, whereas the transfer of early beating clusters into a FBS-enriched cell medium promoted the pacemaker-like phenotype, at the expense of working CMs [74,75]. Numerous researchers employed hiPSC-CMs to investigate different CVDs, as an example Benzoni and colleagues used hiPSC-CMs as a model for a familial form of AF to investigate electrophysiological alterations and identify triggering mechanisms. Patch-clamp recordings detected a higher beating rate, a prolonged action potential duration and a gain of function in calcium L-type current and pacemaker current in AF hiPSC-CMs compared to controls. Furthermore, under stressful conditions, AF hiPSC-CMs displayed a bigger amplitude of delayed after-depolarizations and more frequent ectopic beats. These alterations, due to the genetic background, contribute to create the conditions to develop arrhythmias [76]. This paper is an example of how hiPSC-CMs can recapitulate a disease from its onset. Indeed, the genetic defect in AF hiPSC-CMs initially leads to calcium currents increase, as observed by the authors, while it is known that AF late phases are characterized by their decrement [77]. In this view, an initial calcium overload can trigger maladaptive modifications in atrial protein expression, including Ca^++^- regulating proteins. Another work proposed hiPSC-CMs to study a familial HCM caused by an autosomal missense mutation on *MYH7* gene. This model recapitulated the HCM phenotype: increase in cell size, multinucleation, calcium dysregulation, elevation of β-myosin/α-myosin ratio, expression of ANP and, after 40 days since cardiac commitment, upregulation of hypertrophic-related genes, such as *GATA4*, cardiac muscle troponin 2 (*TNNT2)*, ventricular/atrial myosin regulatory light chain 2 (*MYL2)* and *MYH7*. Researchers demonstrated that pharmacologic treatment of HCM hiPSC-CMs with the L-type Ca^++^ channel blocker verapamil solves Ca^++^ dysregulation, preventing other HCM phenotypic hallmarks and suggesting the role of calcium in the pathogenesis of the disease [78]. hiPSC-CMs have also been used to model different types of Long QT Syndrome (LQT) due to specific mutations in calmodulin, demonstrating a promising future perspective in risk stratification and precision medicine [79].

#### 4.2.2. Human Induced Pluripotent Stem Cells-Derived Endothelial Cells

Most cardiomyopathies and electrophysiological disorders directly involve CMs, but, as we saw previously in this review, non-CM cell types are essential in understanding other CVDs. Other cardiovascular cell lineages, such as vascular endothelial and smooth muscle cells, can be obtained from hiPSCs.

hiPSC-derived ECs have been used for understanding of endothelial dysfunction mechanisms, drug screening, and applications in cell therapy. There are three principal approaches to differentiate hiPSCs in ECs: stromal cell co-culture, embryoid bodies, feeder-free monolayer culture exposed to different molecules or growth factors. Different methods have different efficiency, but, regardless of strategy, all hiPSC-ECs present common features, such as endothelial surface marker expression, ability to form a 2D capillary-like tube in vitro and capacity to uptake LDL [80]. In 2016, Sa et al. compared hiPSC-ECs with pulmonary arterial endothelial cells (PAECs) from the same patients suffering from pulmonary arterial hypertension. The two models had similar behaviors in adhesion, survival, migration, tube formation, confirmed by parallel bone morphogenetic protein receptor type 2, collagen IV, kisspeptin 1, and carboxylesterase 1 expression; additionally, both hiPSC-ECs and PAECs showed a positive response to elafin and FK506, two molecules that improve angiogenesis. These results proposed this model as a good candidate for emerging therapy tests [81]. hiPSC-ECs were also used in the investigation of Moyamoya disease and the RNF213 R4810K polymorphism, which makes the carriers more susceptible to this cerebrovascular pathology. hiPSC-ECs deriving from subjects with one or two RNF213 R4810K alleles exhibited minor angiogenic activity compared with wild-type RNF213 hiPSC-ECs and showed a downregulation in securin gene. These findings were coherent with what happened after an overexpression of RNF213 R4810K or after a downregulation of securin in HUVECs, suggesting hiPSC-ECs as a promising vascular in vitro model for Moyamoya disease [82].

#### 4.2.3. Human Induced Pluripotent Stem Cells-Derived Smooth Muscle Cells

Different protocols have been developed for deriving SMCs from hiPSCs. The main general approaches to obtain SMCs from hiPSCs are monolayer culture and embryoid bodies’ method; both of them consist in mesoderm commitment, followed by SMC differentiation [80]. In 2012, Ge Xin et al. generated hiPSC-SMCs from patients with supravalvular aortic stenosis syndrome caused by an insertion in *elastin* gene. This model recapitulated the patients’ phenotype: a defective organization of smooth muscle α-actin filaments, rescued with treatment with recombinant elastin or enhancement of small GTPase RhoA signaling; an elevated proliferation rate, due to higher ERK1/2 activity; a more frequent migration to PDGF [83]. Another work proposed two distinct hiPSC-SMC differentiations from two distinct commitments: paraxial mesoderm cells (PMCs) and neural crest stem cells (NCSCs). These different methods were used for the study of bicuspid aortic valve-related thoracic aortic aneurysm to demonstrate that SMCs derived from NCSCs, but not from PMCs, contributed to the pathogenesis of the disease. Indeed, only hiPSC-NCSC-SMCs showed a reduction in SMC differentiation marker MYH11, in transforming growth factor β (TGF-β) signaling, and exhibited an increase of mTOR signaling which caused impaired contractile function. In addition, researchers tried a treatment with rapamycin, an inhibitor of mTOR, which rescued the impaired contraction phenotype [84].

## 5. Multicellular Models

Over the years, most cell culture studies were limited to essentially one cell type grown on a rigid substrate, precluding the interactions with extracellular matrix (ECM) and different cell types, which cohabit in vivo. A first move in overcoming this limitation is the use of co-cultures, where more than one cell type is brought into contact with each other, in order to take advantage of the reciprocal influence that is lacking in monocultures. The development of novel and advanced technologies has subsequently allowed a further step: the generation of three-dimensional (3D) mono- and co-cultures that attempt to represent the native tissue in its entirety. The relevant thickness and structure, the influence of neighboring cells and the presence of the extracellular matrix provide a more natural 3D environment that improves intercellular organization and crosstalk. This new approach has opened the doors to the advancement of regenerative medicine, drug screening, and disease modeling.

### 5.1. Co-Cultures

The contribution to a disease often is not due to a single cell type, but multiple cells, belonging to the same or another tissue, participate in the manifestation of the phenotype. One way to mimic this condition is to use co-culture, where different cell types share the same culture environment. The aims of co-culture are to investigate the crosstalk between various cells and getting closer to the in vivo situation. Generally, there are two main groups: target cells, which most contribute to the function of the tissue you want to mimic; assisting cells, which create a more complete environment secreting signaling molecules or producing extracellular matrix and help the target cells to adhere, proliferate or differentiate. However, it is not a unidirectional relationship and target cells can work as assisting cells [85]. An example already mentioned is the stem cells differentiation method that involves co-culture with visceral endoderm-like cells [86].

The contact among cells can be indirect or direct. In indirect co-cultures, distinct cell types have in common the medium, but they are cultivated separately, thus the interaction is mediated by soluble factors; direct co-cultures involve a mixture of different cell types in the same culture, allowing a physical contact [85].

Indirect co-cultures are easy to handle and allow the study of cell-cell crosstalk through paracrine signaling. There are two main methods: conditioned media and transwell co-cultures. Conditioned media approach consists in the transfer of the assisting cells supernatant, rich in soluble factors, into the target cells culture [87]. One of the most studied CVD, exploiting conditioned media, is atherosclerosis, owing to the inflammation of the vessels. Maione et al. exposed primary VSMCs from atherosclerotic patients to conditioned media of activated CD14^+^ cells to investigate the role of CaMKII. Activated CaMKII in macrophages and monocytes stimulated production of cytokines that inhibited CaMKII activity in VSMCs, with the consequent decrease of their proliferation [88]. In transwell co-cultures, both cells types are present in the same environment, but a distant permeable membrane filter inserted in the plate avoids a direct contact. Initially, the different cell types are cultured separately and, secondly, when chosen conditions are reached, they are joined. The filter can variate in size or composition, to satisfy researcher needs [87]. Sebastião and colleagues pointed out an in vitro human model of myocardial IR injury, generating a transwell co-culture with human cardiac progenitor cells (hCPCs) and hiPSC-CMs. The model recapitulated IR phenotype, demonstrating the importance of the co-existence of these two cell types and the role of cytokines: hiPSCs-CMs death due to IR injury is prevented in co-culture conditions, thanks to the paracrine action of hCPCs, cultivated on a semipermeable polyester membrane inside the same plate [89].

Direct co-cultures permit a higher level of communication: a physical cell-cell or cell- extracellular matrix contact. These can be two-dimensional or three-dimensional, and a separate chapter will be dedicated to the latter (Section 5.2). The importance of this intimate interaction is clear in Wallace and colleagues’ work. In the contest of atherosclerosis, researchers measured adhesion molecules expression in primary ECs after TNF-α treatment, which mimicked atherosclerosis inflammation condition. In direct co-culture with primary SMCs, ICAM-1, VCAM-1, and E-selectin surface proteins showed a significant reduction in ECs exposed to TNF-α. Direct co-culture with fibroblasts, indirect transwell co-culture with SMCs and ECs monoculture did not exhibit a decrease of EC adhesion molecules, whereas co-culture with ECs and ECM produced by SMCs displayed a light reduction of these proteins, suggesting that a close contact between ECs and SMCs is required to inhibit TNF-α action [90].

### 5.2. 3D Cultures

In order to better represent the complex three-dimensional (3D) structure and physiology of heart and vessels, several groups dedicated their work to the development of cardiovascular 3D models. With cardiac 3D models, researchers attempt to achieve different requirements, among which appropriate membrane channels expression, functional gap junctions, well-organized sarcomeres, correct alignment of CMs and rich vascularization [91,92]. A crucial role for a better yield of the native tissue is played by bioreactors, which simulate cardiac physiological environment, for example setting the flow of oxygen, cytokines, and nutrients and through the simulation of electromechanical forces. It has been demonstrated that both mechanical conditioning and electrical stimulation lead to a more mature cardiac construct and enhance conductive and contractile properties [93,94]. At the same time, medium perfusion and the related controlled gradient formation permit a spatial control of tissue environment, imitating capillary vascularization efficiency, that is one of the aims of microfluidic platforms [95,96]. The techniques used for the generation of 3D cardiac tissue are multiple and different; over the years they have been refined and a mixture of cardiovascular cell types are usually involved. The most general difference among 3D engineered tissue approaches is the presence of a scaffold.

#### 5.2.1. Scaffold-Free 3D Cultures

Many researchers have developed 3D tissue constructs without the application of an exogenous scaffold, a strategy that improves cellular communication through a closer contact among cells. One of the simplest methods consists in the spontaneous aggregation of cardiac cells under the right conditions, resulting in a cardiac spheroid by self-assembly. The formation of spheroids can occur through hanging drops method, culture on non-adhesive surfaces, rotation systems, microfluidic channels [97,98]. The secretion of ECM by cells in spheroid cultures and the possibility of co-culturing human CMs, cardiac fibroblasts, and coronary artery endothelial cells allow the generation of a vascularized cardiac spheroid that well recapitulates human heart microenvironment, as described in Campbell and colleagues work [99]. More generally, spheroids can be named microtissues, especially when they do not exhibit a spherical shape or they are cultured on adherent substrates [97]. An alternative approach for generating scaffold-free 3D tissues is the formation of cell sheets: CM monolayers are cultured on coatings, stable at 37 °C and that dissolve at room temperature, allowing the detachment of the whole monolayers, which are stacked one upon another to form a thicker cardiac tissue. Among layers, cells establish close interconnections with gap junctions, which let biochemical and electrical exchanges, establishing contractile function. When ECs layers are placed between CM layers, the vascularization is improved, as it happens generating heterogeneous layers including both CMs and ECs, partially solving the problems concerning low perfusion of oxygen and nutrients [100,101].

#### 5.2.2. Scaffold-Based 3D Cultures

Typically, a scaffold serves to support the 3D architecture of the tissue and to employ some appropriate features, including optimal adhesion, migration, growth, organization, differentiation, and function of cells. Scaffolds can originate from synthetic polymers, such as polyglycolic acid (PGA), poly-e-caprolactone, polylactic acid (PLA) or from natural polymers, such as collagen, fibrin, Matrigel [92]. Different types of scaffold with different properties are reported in literature: sponge-like scaffolds, as described by Caspi et al., who cultured hESC-CMs and -ECs on porous sponges composed of synthetic polymers (50% PLA, 50% PGA) to recreate a vascularized myocardium; fibrous scaffolds, used in Kenar et al. work, where MSCs were seeded on a multi-layered microfibrous mat to align a myocardial construct; scaffolds in the form of a gel, as reported by Acun et al., who encapsulated hiPSC-derived CMs and ECs in hydrogels [102,103,104]. The suspension of cells in hydrogels is the most commonly used 3D scaffold-based model. Hydrogels composition is chosen by researcher and depends on the purpose of the investigation: they are prepared with hydrophilic polymers, natural or synthetic, can absorb a great amount of fluids and generate a cross-linked matrix that can mimic the native ECM [105]. Besides artificial matrices, it is also possible to obtain a completely natural scaffold from decellularized tissue deriving from animal hearts or from plants, subsequently repopulated with CMs and ECs [106,107]. This approach can be adapted emulsifying the remaining natural ECM and reconstituting it as decellularized myocardial matrix hydrogel [108].

#### 5.2.3. Printed 3D Cultures

A separate discussion is reserved for 3D printing techniques, which have the ability of producing a 3D object of any shape from a digital model. This technology can be applied in two ways: the manufacture of a scaffold or a mold in which cells are subsequently seeded, and the more advanced bioprinting strategy, which involves the use of cell suspension as part of the “ink”, defined bioink [109]. In the first case, scaffolds with complex geometric structures can be printed using synthetic or natural biocompatible materials, as described by Gao et al., who generated a native-like cardiac ECM architecture with a solution of gelatin methacrylate as ink, then filled up with hiPSC-CMs, -ECs and -SMCs, obtaining a cardiac beating muscle patch [110,111,112]. On the other side, novel bioink-based approaches allow direct printing of cells or spheroids suspended in polymer scaffolds, hydrogels, or decellularized ECM, manufacturing cardiovascular tissues. As usual, scaffold composition and cell types involved in the printing procedure can be adjusted in order to mimic at best the native tissue and the pathologic phenotype in disease modeling. For instance, in 2012, Gaetani and colleagues printed human fetal CM progenitor cells resuspended in alginate gel, whereas in 2017, Pati et al. generated a cardiac tissue model using decellularized ECM and MSCs [113,114].

#### 5.2.4. 3D Organ-On-A-Chip

Currently, 3D constructs mimicking cardiovascular tissues have been implemented with the concept of organ-on-a-chip (OOC), designed to obtain a completely integrated, miniaturize cardiovascular system. Methods to engineer heart-on-a-chip or vessel-on-a-chip are numerous and various, but are predominantly based on microfluidic technology and commonly involve culture of cardiovascular cells on polydimethylsiloxane (PDMS) devices, which have the advantages of being flexible and optical transparent [115]. The native tissue environment is recreated with architectural structure, contractile properties, simulation of vessel flow, through controllable shear stresses imitating hemodynamic forces, uniaxial cyclic strains and application of electric fields generating a synchronous beating activity, perfusable microchannels for efficient delivery of oxygen and nutrients [116,117,118,119]. For example, Marsano and colleagues’ work demonstrated the importance of a mechanical stimulation for the maturation of CMs, fabricating a microfluidic platform for the generation of a 3D microcardiac construct, which recapitulated the electromechanical properties of the heart [120]. Campisi et al. recreated the human brain blood barrier by self-assembly of hiPSC-ECs co-cultured with brain pericytes and astrocytes on a microfluidic platform, which permitted the formation of a perfusable microvascular network with low permeability and transport selectivity [121].

#### 5.2.5. Examples of Applications of 3D Cultures

Several diseases have been investigated using the 3D model approaches mentioned above, involving the co-culture of different cardiovascular cellular types. For example, a self-assembled scaffold-free microtissue containing CMs, endothelial cells and cardiac fibroblasts deriving from hiPSCs was carried out by Giacomelli et al. to explore the role of cardiac fibroblasts in hiPSC-CMs maturation and their implication in ACM. They demonstrated that cardiac fibroblasts improved the structural, electrical, and metabolic maturation of hiPSC-CMs through a mechanism involving Cx43 and cyclic adenosine monophosphate (cAMP); furthermore, they showed that the presence of ACM cardiac fibroblasts is sufficient to manifest ACM phenotype in the whole microtissue, otherwise composed of NON-ACM cells [122]. The cell-sheet method served Kawatou and colleagues for the development of a 3D model that recapitulated the torsade de pointes (TdP) arrhythmia. Cardiac tissue sheets, composed of both CMs and mesenchymal stromal cells, deriving from hiPSCs or composed of only hiPSC-CMs, were exposed to I_Kr_ channel blockers in order to induce the typical tachyarrhythmia of TdP. The researchers found that both cellular heterogeneity and a certain thickness of 3D structure were required to observe TdP phenotype [123]. Chen et al. generated a myocardial IR injury model encapsulating hiPSC-CMs in a hydrogel scaffold composed of collagen type I and fibrinogen. The resultant cardiac tissue, after IR condition simulation, recapitulated crucial aspects of IR injury, such as alterations in cell viability, mitochondrial membrane permeability, reactive oxygen levels, intracellular pH [124]. A scaffolded model of atherosclerosis was developed by Robert and collaborators, who produced a vessel-like structure from human umbilical cord-derived myofibroblasts and HUVECs, utilizing a biodegradable tubular scaffold matrix made-up with PGA meshes. The activation of the engineered endothelium provoked an increase in endothelial and sub-endothelial accumulation of LDL and HDL, in monocytes adhesion and in their transmigration to the intima [125]. A synthetic filamentous matrix covered with hiPSC-CMs from LQT type 3 patients was developed to mimic the aligned ventricular myocardium in order to investigate LQT type 3 abnormalities. Researchers found that the stiffness of the engineered filaments influenced contractility and electrophysiological response to different drugs, highlighting the crucial importance of the myocardial matrix mechanical properties [126]. In 2018, van der Valk et al. generated a 3D model of calcific aortic valve disease (CAVD), bioprinting 3D hydrogels with encapsulated primary human valve interstitial cells, which mimicked native aortic valve mechanical properties. The treatment of hydrogels with osteogenic media induced microcalcifications accumulation, collagen secretion, and upregulation of metalloproteases, replicating the progression of CAVD in vivo [127]. OOC technology served to model the mitochondrial cardiomyopathy of Barth syndrome by the generation of engineered heart-on-a-chip with hiPSC-CMs. Researchers performed metabolic and functional analysis to show that *Tafazzin* gene mutation leaded to contractile impairments and overproduction of ROS, and that gene replacement worked to rescue the pathological phenotype [128]. Another work proposed a model of cardiac-fibrosis-on-a-chip co-culturing cardiac fibroblasts and hiPSC-CMs on a microfabricated device: exposure to TGF-β triggered the typical fibrosis followed by heart failure, but anti-fibrotic drugs restored the pathological phenotype, demonstrating the potential of OOC technology for disease modeling and drug discovery [129].

## 6. Discussion

**Figure 1:**
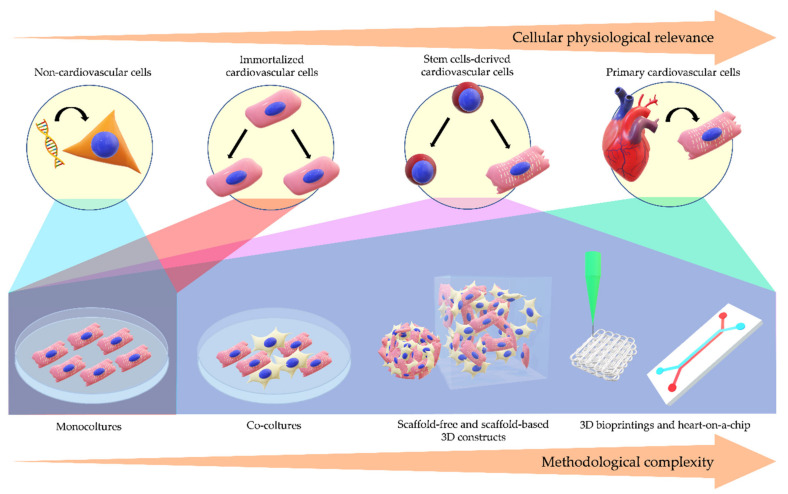
Disease Modeling Complexity. A schematic overview of the increasing complexity of the main cell models commonly used for cardiovascular diseases (CVDs) and their relative availability for increasingly complex methodologies.

In this review, we provided an overview from the simplest to the most sophisticated human cellular models for cardiovascular research (Figure 1).

The use of heterologous systems for the genetic manipulation of specific genes has dramatically fueled scientific research, allowing the comprehension of the functions of these genes in their healthy or pathologic version. Such observations occur in an isolated and highly reproducible context allowing the comparison of different mutations of the same protein in the same genetic setting. Nevertheless, these models lack the genetic and epigenetic backgrounds of the patients, which often influence the resulting phenotype, and are not useful for polygenic diseases or for acquired disorders. In general, any CVD in its entirety could not be studied on heterologous systems, which lack the molecular scenario and morphologic features of cardiovascular cells.

This is also true for patient-derived non-cardiovascular cells, useful when they express the same mutant proteins associated to CVDs. These cells can represent an additional model for mechanistic studies and drug discovery in large patient cohorts, thanks to their easy accessibility with non-invasive sampling techniques. However, despite the advantage of the direct derivation from patients, their non-cardiac origin restricts the range of investigation.

As previously mentioned, primary cell models are the most intuitive method for studying a CVD. However, adult cells carrying patient’ genetic background are poorly available. Moreover, samples are often obtained from subjects in advanced state of morbidity, making mechanistic studies hard to be finalized. Some primary cells, especially CMs, possess a short lifespan and cannot be amplified in vitro, limiting the number of experiments and the variety of assays.

Over the years, researchers tried to overcome these obstacles exploiting the expression of oncogenes to immortalize post-mitotic cells, forcing their cycle re-entry and the consequent proliferation. These attempts started from animal immortalized cardiac cells, such as mouse HL-1, to human with AC 16 line [50,130]. The human ventricular AC 16 cells present the great benefit to be proliferative CMs, but, owing the SV40 transformation, they show some defects, such as expression of atrial markers, a pre-contractile stage, lack of many inward and outward currents, which do not start adequate action potentials, and they do not entirely recapitulate the cellular context of CMs [50].

All these issues were partially solved with the introduction of hESCs. Their ability to proliferate for extended periods in culture and to differentiate towards tissue derivatives from the three germ layers, together with the possibility to generate recombinant hESC lines with different mutations by gene targeting techniques, promised a revolution in the comprehension of developmental processes, pathological molecular mechanisms, drug discovery and cell therapy. In the years following their isolation, numerous protocols for efficient differentiation were developed and refined, but ethical problems associated with the use of embryos for hESCs derivation and hiPSCs discovery restricted the use of hESCs as a disease model. hiPSCs made cardiac and vascular cells even more accessible, thanks to the possibility to generate pluripotent stem cells similar to hESCs, by reprogramming somatic cells from healthy donors and from patients affected by specific CVDs. hiPSCs allow to investigate genotype-phenotype relationships in monogenic, polygenic and genetically unknown conditions. A critical argument is the choice of controls: in order to avoid as much as possible the genetic variability between patients and control cells, the best option is the generation of an isogenic control, correcting the mutation in the patient hiPSC line through gene targeting approach [131]. To date, cell types deriving form pluripotent stem cells show an immature phenotype. This is particularly true for CMs, whose maturation is now improved with various methods, including anisotropic signals, electromechanical conditionings, biochemical modulations, multiple cell type co-cultures [132,133]. Co-cultures allow the generation of a more complex tissue, which better mimics the microenvironment of origin, thanks to the influence of extracellular matrix and cells that normally coexist in the tissue. New technologies have allowed to recreate this microenvironment in 3D constructs, mimicking the cell-cell and cell-matrix mechanics and structural organization observed in vivo like never before. In particular, OOC, together with 3D bioprinting technics, emerged as the most advanced methods to develop 3D tissue models in vitro with opportune anatomical and pathophysiological features [134]. The resemblance to human organs reached with these novel strategies allows a more complete comprehension of pathogenic mechanisms and provides a more efficient tool for drug discovery. Furthermore, the recent concept of body-on-a-chip aims to reflect the interaction among organs in vivo, combining multiple OOC in a unique integrated system for various potential biomedical applications, such as disease modeling, drug discovery, biomarker detection [135,136,137]. In this way, it could be possible to observe the role of other organs, such as nervous or respiratory systems, which are closely interconnected with heart and vessel physiology, and sometimes participate to pathogenesis of specific CVDs.

The generation of advanced and easily reproducible disease models, along with epigenetics, next generation sequencing and ‘omics’ studies, improves the comprehension of CVDs, leading to the development of personalized medicine in vitro, for both individuals and patients’ sub-populations [138]. Different conditions, such as cardiac arrhythmias, cardiomyopathies and vascular hypertension, may show a similar phenotype despite different specific causes, with consequent distinct drug response. In these cases, differential diagnosis, risk stratification, and personalized treatments become necessary, in order to refine the understanding of genotype-phenotype relation and to implement the efficacy of clinical research [139,140,141,142].

As previously mentioned, the fabrication of engineered 3D tissues, increasingly sophisticated and close to reality, provides flexible tools that also aims to replace various animal-based studies, reducing their use in laboratory to model CVDs. At the same time, in vivo models are necessary for the validation of 3D constructs before applying them on humans, since a future prospect is their application in regenerative medicine as biologically relevant patches for tissue repair [143,144,145]. Moreover, pluripotent stem cells could become a tool for clinical applications: a lot of studies on cardiac regenerative therapy were carried out, showing improvements in cardiac function after transplantation in animals [146,147,148]. Nevertheless, some factors must be considered, indeed, in vivo, hESCs cause an immune response and require immunosuppression, unlike hiPSCs, which can be derived from the specific patient. Moreover, because of the immature phenotype, the differentiation has to be improved, in order to obtain only terminally differentiated cells, avoiding teratoma formation [149].

Obviously, further studies are needed before the application to the clinical practice will be a reality. Emerging technologies will allow in the future an increasingly precise understanding of the pathophysiology of CVDs and consequent therapeutic advancements.

## 7. Conclusions

An overview is offered of the main human-derived in vitro models used in cardiovascular translational research, focusing on advantages and disadvantages of each, and illustrating examples of their applications to recapitulate disease pathogenesis, as a support to human scientific research and medical science on the topic (Table 1). The mentioned tools can indeed be exploited for molecular mechanistic studies, drug screening, and personalized medicine. Extensive efforts are constantly made to refine the approaches by improving cellular physiological relevance as well as methodological diversity in order to push disease modeling to a major level of complexity while keeping the simplicity of readouts necessary to understand the disease pathogenesis.

## Figures and Tables

**Table 1:** Summary of mentioned examples of human cell modeling for cardiovascular diseases and the relative clinical relevance.

Cell Models	Cardiovascular Diseases	Clinical Relevance	References
HEK 293	Familial sinus bradycardia	Pathogenic mechanism	[6]
	Arrhythmogenic Cardiomyopathy	Pathogenic mechanism	[8]
BMCs	Arrhythmogenic Cardiomyopathy	Pathogenic mechanism	[10]
	Heart failure	-	[11]
CMs	Heart failure	Pathogenic mechanism	[14]
	Hypertrophic Cardiomyopathy	Drug discovery	[16,17]
C-MSCs	Myocardial ischemia	Pathogenic mechanism	[24]
	Arrhythmogenic Cardiomyopathy	Pathogenic mechanism/Involvement in disease pathogenesis	[25]
c-kit ^+^ C-MSCs	Atrial Fibrillation	Pathogenic mechanism	[31]
FAPs	Arrhythmogenic Cardiomyopathy	Involvement in disease pathogenesis	[35]
ECs	Hypertension	Pathogenic mechanism	[39,43]
	Atherosclerosis	Pathogenic mechanism/Involvement in disease pathogenesis	[41]
		Pathogenic mechanism	[42]
VSMCs	Hypertension	Involvement in disease pathogenesis	[48]
		Pathogenic mechanism	[49]
	Atherosclerosis	Differentiation phenotypes	[46]
		Pathogenic mechanism/Drug discovery	[47]
AC 16	Hypertrophic Cardiomyopathy	Drug discovery	[51]
	Myocardial ischemia	Pathogenic mechanism	[52]
hESC-CMs	Hypertrophic Cardiomyopathy	Pathogenic mechanism	[64,65]
hiPSC-CMs	Atrial Fibrillation	Pathogenic mechanism	[76]
	Hypertrophic Cardiomyopathy	Drug discovery	[78]
	Long QT Syndrome	Pathogenic mechanism/Drug discovery	[79]
hiPSC-ECs	Hypertension	Drug discovery	[81]
	Moyamoya disease	Pathogenic mechanism	[82]
hiPSC-SMCs	Supravalvular aortic stenosis syndrome	Pathogenic mechanism	[83]
	Bicuspid Aortic Valve-related Thoracic Aortic Aneurysm	Pathogenic mechanism/Drug discovery	[84]
VSMCs and CD14^+^ cells co-culture	Atherosclerosis	Pathogenic mechanism	[88]
hCPCs and hiPSC-CMs co-culture	Myocardial ischemia	Regenerative therapy mechanisms	[89]
ECs and VSMCs co-culture	Atherosclerosis	Pathogenic mechanism	[90]
hiPSC-CMs, hiPSC-ECs and hiPSC-cardiac fibroblasts: scaffold-free 3D microtissue	Arrhythmogenic Cardiomyopathy	Pathogenic mechanism	[122]
hiPSC-CMs and MSCs: cell sheet	Torsade de pointes	Pathogenic mechanism/Drug response	[123]
hiPSC-CMs: scaffold-based 3D tissue	Myocardial ischemia	Pathogenic mechanism	[124]
ECs: scaffold-based 3D tissue	Atherosclerosis	Pathogenic mechanism	[125]
hiPSC-CMs: scaffold-based 3D tissue	Long QT syndrome	Pathogenic mechanism/Drug response	[126]
3D-bioprinted valve interstitial cells	Calcific aortic valve disease	Pathogenic mechanism	[127]
hiPSC-CMs: heart-on-a-chip	Mitochondrial cardiomyopathy of Barth syndrome	Pathogenic mechanism	[128]
hiPSC-CMs and cardiac fibroblasts: heart-on-a-chip	Cardiac fibrosis	Drug discovery	[129]

## Abbreviations

3D	three-dimensional
ACM	arrhythmogenic cardiomyopathy
AF	atrial fibrillation
Ang II	angiotensin II
ANP	atrial natriuretic peptide
BMC	buccal mucosa cell
BMP2	bone morphogenetic protein 2
BNP	B-type natriuretic peptide
CaMK II	Ca^++^/calmodulin-dependent protein kinase II
cAMP	cyclic adenosine monophosphate
CASM	smooth muscle cells from coronary artery
CAVD	calcific aortic valve disease
C-kit	KIT proto-oncogene receptor tyrosine-protein kinase
CM	cardiomyocyte
CMPC	cardiac mesenchymal progenitor cell
C-MSC	cardiac mesenchymal stromal cell
CRP	C-reactive protein
CTEPH	chronic thromboembolic pulmonary hypertension
CVD	cardiovascular disease
Cx40	connexin 40
Cx43	connexin 43
EC	endothelial cell
ECM	extracellular matrix
ERBB	erythroblastic oncogene B
ERK1/2	extracellular signal-regulated kinase ½
ESC	embryonic stem cell
FAP	fibro/adipocyte progenitor
FBS	fetal bovine serum
FD	Fabry disease
Gb3	globotriaosylceramide
GLA	α-galactosidase A
GSK3β	glycogen synthase kinase 3β
GTPase RhoA	GTPase Ras homolog family member A
HAEC	human aortic endothelial cell
HA-VSMC	human aortic vascular smooth muscle cell
HCM	hypertrophic cardiomyopathy
HCN4	potassium/sodium hyperpolarization-activated cyclic nucleotide-gated channel 4
hCPC	human cardiac progenitor cell
HDL	high density lipoprotein
HEK	human embryonic kidney
hESC	human embryonic stem cell
hiPSC	human induced pluripotent stem cell
HLA-DR	human leukocyte antigen—DR isotype
HMGB1	high-mobility group box-1
HUVEC	human umbilical vein endothelial cell
ICAM1	intercellular adhesion molecules 1
IHF	ischemic heart failure
I_K1_	inward rectifier K^+^ current
I_KUR_	ultra-rapid delayed rectifier K^+^ current
iPSC	induced pluripotent stem cell
IR	ischemia/reperfusion
Iso	isoproterenol hydrochloride
I_to_	transient outward K^+^ current
JNK	c-Jun-N-terminal kinase
LDL	low-density lipoproteins
LOX1	lectin-like oxLDL receptor 1
LQT	long QT syndrome
MI	myocardial infarction
miR-133	microRNA-133
MMP	matrix metalloproteinase
MSC	mesenchymal stromal cell
mTOR	target of rapamycin
MYH11	myosin heavy chain-11
MYH7	myosin heavy chain-7
MYL2	myosin light chain-2
NANOG	homeobox protein NANOG
NCSC	neural crest stem cell
NF-κB	nuclear factor k-light-chain-enhancer of activated B cells
NRG	neuregulin
Oct-4	octamer-binding transcription factor 4
OOC	organ-on-a-chip
p38-MAPK	p38 mitogen-activated protein kinase
PAEC	pulmonary arterial endothelial cell
PASMC	pulmonary artery smooth muscle cells
PDGF	platelet-derived growth factor
PDGFRα	platelet-derived growth factor receptor α
PDMS	polydimethylsiloxane
PG	plakoglobin
PGA	polyglycolic acid
PKC-α	protein kinase C-α
PLA	polylactide acid
PMC	paraxial mesoderm cell
PQQ	pyrroloquinoline quinone
PRR	pro-renin receptor
RAS	renin-angiotensin system
ROS	reactive oxygen species
RyR	ryanodine receptor
S1PR1	sphingosine-1-phosphate receptor 1
Sox2	sex determining region Y-box 2
SSEA-4	stage-specific embryonic antigen 4
SVSM	smooth muscle cells from saphenous vein
TdP	torsade de pointes
TGF-β	transforming growth factor β
TNF-α	tumor necrosis factor-α
TNNT2	troponin 2
VCAM1	vascular cell adhesion protein 1
VSMC	vascular smooth muscle cell
WT	wild type
YAP1	yes-associated protein 1
β1AR	β1-adrenergic receptor
β-MHC	β-myosin heavy chain
